# Building a bio-based industry in the Middle East through harnessing the potential of the Red Sea biodiversity

**DOI:** 10.1007/s00253-017-8310-9

**Published:** 2017-05-20

**Authors:** Jens Nielsen, John Archer, Magbubah Essack, Vladimir B. Bajic, Takashi Gojobori, Ivan Mijakovic

**Affiliations:** 1 Department of Biology and Biological Engineering, Chalmers University of Technology, Kemivägen 10, SE412 96 Gothenburg, Sweden; 2 Novo Nordisk Foundation Center for Biosustainability, Technical University of Denmark, 2800 Kgs. Lyngby, Denmark; 3 Science for Life Laboratory, Royal Institute of Technology, SE17121, Solna, Sweden; 4 Pathogen Genomics Laboratory, Biological and Environmental Sciences and Engineering (BESE) Division, King Abdullah University of Science and Technology (KAUST), Thuwal, 23955-6900 Kingdom of Saudi Arabia

**Keywords:** Metabolic engineering, Synthetic biology, Industrial biotechnology, Cell factories, Metagenomics

## Abstract

The incentive for developing microbial cell factories for production of fuels and chemicals comes from the ability of microbes to deliver these valuable compounds at a reduced cost and with a smaller environmental impact compared to the analogous chemical synthesis. Another crucial advantage of microbes is their great biological diversity, which offers a much larger “catalog” of molecules than the one obtainable by chemical synthesis. Adaptation to different environments is one of the important drives behind microbial diversity. We argue that the Red Sea, which is a rather unique marine niche, represents a remarkable source of biodiversity that can be geared towards economical and sustainable bioproduction processes in the local area and can be competitive in the international bio-based economy. Recent bioprospecting studies, conducted by the King Abdullah University of Science and Technology, have established important leads on the Red Sea biological potential, with newly isolated strains of Bacilli and Cyanobacteria. We argue that these two groups of local organisms are currently most promising in terms of developing cell factories, due to their ability to operate in saline conditions, thus reducing the cost of desalination and sterilization. The ability of Cyanobacteria to perform photosynthesis can be fully exploited in this particular environment with one of the highest levels of irradiation on the planet. We highlight the importance of new experimental and in silico methodologies needed to overcome the hurdles of developing efficient cell factories from the Red Sea isolates.

## Introduction

Humans have exploited microorganism for production of fermented food products for thousands of years, but with the identification and isolation of microbial species responsible for specific fermentation process, e.g., production of beer and wine, the path was opened for wider use of microorganisms for production of chemicals. This led Chaim Weismann to develop a fermentation process for production of acetone-butanol-ethanol during World War 1 (Weizmann 1915), a process that was used for about 50 years to produce acetone and is today being revived for production of 1-butanol that can be used as a chemical building block and as a biofuel. A few years later, the filamentous fungus *Aspergillus niger* was identified to have the ability to produce citric acid at conditions of very low pH, and this led to the development of the first large-scale aerobic fermentation process for production of citric acid (Currie 1917). Today this process is still in use for production of citric acid that is used in soft drinks and for food preservation (Papagianni 2007). Another landmark in industrial fermentation was the development of a large-scale process for production of penicillin, which was discovered by Alexander Fleming in 1929 (Fleming 1944). It took several years from the original discovery to development of an industrial process, primarily due to belief that penicillin could be synthesized chemically. However, during World War 2 the War Production Board in the USA decided to endorse the fermentation route, which resulted in production of the first large-scale batches of penicillin in 1943. The development of an industrial scale fermentation process for production of penicillin was challenging. Knowledge from citric acid production at large scale could be used, but as penicillin production is not at low pH it was necessary to develop the technology for sterilization of production fermenters and ensure provision of large amounts of sterile air required for the aerobic production process. Following World War 2, the same technology used for production of penicillin could rapidly be applied for production of many other antibiotics that were discovered in the 1950s and 1960s. Development of the abovementioned three production processes each represent technological breakthroughs that today supports a billion USD industry where microorganisms are used for production of pharmaceuticals, enzymes, biofuels, chemicals, food, and feed ingredients, and we will later provide a brief review of products currently produced by microbial fermentation. Even though the production of recombinant proteins used as pharmaceuticals represent a major contribution of this market value, with an estimated value of about USD 100 billion (Nielsen 2013), the production of fuels and chemicals is increasing rapidly with a market value of USD 32 billion in 2010 and estimated to be above USD 75 billion in 2017.

There are three key drivers for the fuel and chemical industry to shift towards bio-based production: (1) The opportunity to produce new molecules that have improved properties compared with currently available chemicals. A good example of this, as will be discussed later, is lactic acid to be used for production of polylactic acid (PLA). PLA has a number of properties as a polymer that makes it well suited for wide applications, including fabrics to be used in sports clothes and biodegradable packing materials (Lunt 1998). (2) The opportunity to produce chemicals cheaper than through traditional routes. Even though the chemical industry has been extremely successful in producing a wide range of chemicals from oil, some chemicals are hard to produce at low costs through cracking and catalysis. A good example of this is 1,3 propanediol, as also will be discussed below, that can be used to produce Sorona®, a valuable polymer that is used in fabrics and carpets (Ritter 2003). (3) The opportunity to produce chemicals with a reduced environmental footprint. Many new biotech processes have a lower environmental footprint than traditional chemical synthesis, not only in terms of carbon dioxide emission but also in terms of water and energy usage. Furthermore, through the use of plant materials, either crops or biomass, there is a sustainable availability of the feedstock used, which makes the production independent of oil availability (Shahzad et al. 2013).

**Fig. 1:**
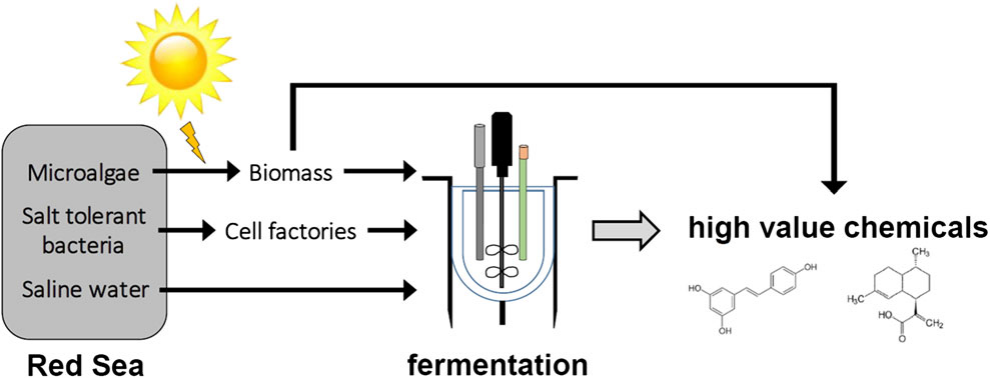
The Red Sea is a rich and untapped ecosystem for bio-based production of high-value chemicals. Microalgae and/or cyanobacteria that can grow in the saline and high sun-intensive environment can be used for generation of biomass to be used for microbial fermentation. These can be grown in farms at the shore right next to biorefineries. It may be possible to extract some high-value chemicals directly from the microalgae, with the remainder of the biomass being used for microbial fermentation. In order to reduce cost, the biomass should not be extracted from isolated saline water, but it should be hydrolyzed in suspension. This will require isolation of novel, salt-tolerant enzymes, e.g., to be produced by *Bacillus* species isolated from the Red Sea. Hydrolyzed biomass, suspended in saline water, will be used for microbial fermentation to produce high value chemicals. Cell factories for these processes will also be isolated from the Red Sea. Running the fermentation process with saline water will reduce (1) the risk of contamination, which will reduce costs for sterilization, and (2) the cost incurred from energy consumption for saline water desalination

There are, however, several challenges for developing a bio-based process for chemical production: (1) Bio-based production requires development of a cell factory that can efficiently convert the feedstock, typically sugar, to the product of interest. This requires extensive engineering of the cellular metabolism, which is challenging due to extensive regulation of metabolism in living cells. (2) It is important to have continuous access to a feedstock that can be used for production for microbial fermentation. (3) Development of a bioprocess that is matched with the cell factory and that can ensure low cost production of the chemical at large scale. Here we will briefly review current bio-based production of fuels and chemicals and we will thereafter discuss the opportunities for developing bio-based production of fuels and chemicals in the Middle East. Our discussion will follow the concept illustrated in Fig. 1.

## The current bioindustry

As mentioned above, the market for renewable chemicals is growing rapidly and a list of some of the major products is provided in Table 1. One observation from this list is that relatively few cell factories are used for production of a quite wide range of chemical compounds, and these widely used cell factories are typically referred to as platform cell factories (Nielsen and Keasling 2016). The main reason for this is the challenge of developing an efficient cell factory, and industries therefore prefer to use cell factories for which there is a large knowledge base, both in terms of genetics and physiology and in terms of industrial scale production. The largest market for fermentation products, by far, in terms of volume is ethanol, which is used as a biofuel. Ethanol is predominantly produced in the USA and in Brazil from corn and sugar cane, respectively. This process has become the benchmark for many other industrial fermentation processes, both in terms of defining bioprocessing costs and in terms of defining requirements for the cell factory. It is a relatively low-cost process as there is no need for aeration. The rapid production of ethanol prevents, to a large extent, contamination of the bioreactor, which means that costs for sterilization can be reduced or even completely eliminated. Based on Fig. 1, we envisage that by using highly saline water from the Red Sea combined with salt-tolerant cell factories, it will similarly be possible to establish bioprocesses that have similarly reduced (or no) costs for sterilization of the feedstock. Below we will discuss some of the challenges associated with development of novel bioprocesses and thereafter describe a few bioprocesses introduced in recent years due to either the ability to reduce costs and environmental footprint or to provide new chemicals.

**Table 1:** List of key chemicals produced from renewable sources

Industry segment	Chemical	Cell factory	Key producers
Fuels	Ethanol	*S. cerevisiae*	Many
	Isobutanol	*S. cerevisiae*	Gevo, Butamax
	Farnesene	*S. cerevisiae*	Amyris/Total
Bulk	1,3 Propanediol	*E. coli*	DuPont
	1,4 Butanediol	*E. coli*	Genomatica/BASF/ Novamont/Cargill
	Lactic acid	pH-tolerant yeast	Cargill
Feed ingredients	Lysine	*C. glutamicum*	Ajinomoto, Evonik, CJ, ADM
	Threonine	*E. coli* *C. glutamicum*	Ajinomoto, Evonik, ADM, Chiel Jedang
Food ingredients	Glutamate	*C. glutamicum*	Ajinomoto
	Citric acid	*A. niger*, *A. wentii*	Cargill, ADM
Pharmaceuticals	Penicillin	*P. chrysogenum*	Many
	7-ADCA^a^	*P. chrysogenum*	DSM
	Artimisinic acid	*S. cerevisiae*	Artepharm, Amyris/Sanofi- Avensis
	Hydrocortisone	*S. cerevisiae*	Sanofi-Avensis
Vitamins etc.	Riboflavin	*A. gossypii*, *B. subtilis*	BASF, DSM
	EPA^b^	*Y. lipolytica*	DuPont
	Resveratrol	*S. cerevisiae*	Evolva
Industrial enzymes	Many	*A. niger, A. oryzae, Bacillus species*	Novozymes, DuPont, DSM
Fine chemicals	Sesquiterpenes	*S. cerevisiae*	Amyris, Evolva

^a^7-aminodesacetoxycephalosporanic acid

^b^Eicosapentaenoic acid

^c^A large number of different industrial enzymes are produced for use in the food, feed and starch industries

^d^Today, several different sesquiterpenes used as perfume ingredients are produced by fermentation

## Challenges in developing new bioprocesses

As mentioned in the introduction, there are three key challenges in connection with developing of a bioprocess: (1) developing of the biocatalyst, or cell factory; (2) ensuring feedstock for the bioprocess; and (3) developing a cost efficient bioprocess. The key challenge in connection with developing a novel bioprocess is the engineering of an efficient biocatalyst, i.e., the cell factory, and typical costs for developing a novel bioprocess amounts to US$50–100 million, whereof a major fraction is for cell factory development (Nielsen and Keasling 2016). Development of a cell factory involves the following steps (Nielsen and Keasling 2016): (1) molecule identification; (2) pathway identification; (3) reconstruction of relevant pathway in chosen cell factory; and (4) optimization of the titer, rate, and yield (TRY) required for a cost competitive process. Molecule identification will typically come as input from the industry segment where the chemical is going to be applied and requires a detailed analysis of market developments as well as the associated intellectual property, i.e., it is possible to obtain freedom to operate in terms of engineering of the cell factory and subsequent production in countries of interest. When a proper molecule has been identified, the next step is to identify if there is a known biological route for biosynthesis of this molecule. With the immense biological variability, it is often possible to find a suitable metabolic pathway leading to the chemical of interest, but in some cases, it may be necessary to develop novel enzymes, which is challenging but feasible. When a pathway has been identified, the genes encoding the individual enzymes will be cloned into the cell factory chosen for the production process. As mentioned earlier, there is a tendency to use one of the few cell factories as production platform, generally referred to as cell factory platforms.

**Fig. 2:**
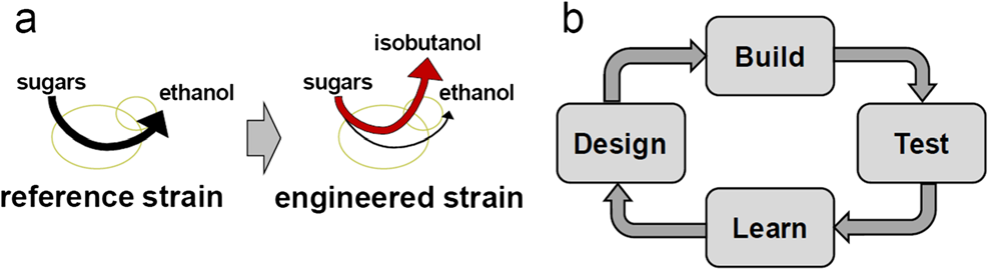
Development of a cell factory. **a** A reference strain, e.g., a strain that can efficiently convert sugars to ethanol, is engineered by first reconstruction of a novel pathway that leads to the product of interest, here illustrated as a *red pathway* towards isobutanol. Thereafter, the cell factory is engineered such that the majority of the sugar is converted to the desired product and no (or little) sugar is converted to ethanol (the original product). The last step is to ensure that the engineered strain has the right titer, rate, and yield (TRY) metrics to allow for cost-competitive production of chemical. **b** In order to reach the TRY targets for industrial implementation, it is generally necessary to perform extensive engineering of the cell factory. This involves several rounds of the so-called Design-Build-Test (DBT) cycle. Here designs are developed based on physiological knowledge or through mathematical modeling and these designs are being built into the strain. The engineered strain(s) are then tested, preferentially at industrial-like conditions. By going through the DBT cycle, much knowledge is gained about the physiology of the cell factory, and it is important to accumulate this knowledge and learn from each design step

There are several reasons why industry prefers to use a few cell factory platforms: (1) Cell factory development typically involves extensive engineering of the cellular metabolism and therefore involves several rounds of the so-called Design-Build-Test (DBT) cycle (Fig. 2). It is therefore advantageous to use extensively studied organisms, like *Saccharomyces cerevisiae* and *Escherichia coli*. These two are arguably the best studied microorganisms, with a wealth of knowledge available, both at the genome level, i.e., extensive omics data, and at the molecular level, e.g., at the enzymology level, and it is therefore easier to engineer these for improving the TRY. However, for some products, these organisms are not well suited and other cell factories are therefore chosen in these cases. For instance, *S. cerevisiae* and *E. coli* are not well suited for high-level secretion of proteins, and Aspergilli and Bacilli are therefore often used as cell factories for production of industrial enzymes. Furthermore, *Corynebacteria* are very efficiently producing and secreting amino acids and are therefore used for production of amino acids and hereof derived products. (2) In order to engineer the cell factory, it is necessary to have good genetic tools. This again favors well-studied organisms like *S. cerevisiae* and *E. coli*, but with the introduction of CRISPR-Cas9 technologies, it is becoming possible to easily engineer many other cell factories. (3) It is important to have organisms that are tolerant to industrial-like conditions. In many cases, industrial fermentations are carried out using complex feedstocks that may cause high osmotic pressure, low pH, and high temperatures. Organisms that have proven to tolerate these conditions will be preferred, as it is generally difficult to engineer the organism to have improved tolerance (generally characterized as complex traits). (4) Finally, it is important to have knowledge on how the cell factory functions at large scale, i.e., does prior knowledge exist about how studies in the laboratory translates to large-scale fermentation processes. It is quite expensive to scale-up a process and it is therefore important to have knowledge from previous scale-up studies with the same cell factory. The move towards the use of a few cell factories is best illustrated by the enzyme industry. Traditionally, many different cell factories were used for production of different enzymes, but today leading companies like Novozymes and DuPont rely on the use of a few cell factory platforms, e.g., *A. niger*, *Aspergillus oryzae*, *Bacillus subtilis* and *Bacillus clausii*. The use of only a few cell factory platforms has allowed the industry to rapidly develop a production process for a new enzyme, as the technology basically becomes plug-and-play where the gene encoding a new enzyme is simply inserted into an efficient enzyme producer, and the resulting cell factory can be transferred to large-scale production following a few tests in the laboratory.

Even though it may be challenging to reconstruct a biosynthetic pathway in a given cell factory, this has now been demonstrated for a wide variety of products (Wang et al. 2012; El Zoeiby et al. 2001; Rugbjerg et al. 2013; Xu et al. 2012; Li et al. 2011; Sun et al. 2010). The choice of cell factory depends on the properties of the product and the pathway to be reconstructed. Thus, for commodity-like products, where high rates are important for the economics of the process, *E. coli* is to be preferred, as illustrated by the use of this cell factory for the production of 1,3-propanediol (Emptage et al. 2003; Diaz-Torres et al. 2000) and 1,4 butanediol (Yim et al. 2011). However, if the product is an organic acid, it is preferential to use an organism that can tolerate low pH as the product can hereby be produced directly in its acid form. *A. niger* is therefore used for production of citric acid, and a low pH-tolerant yeast was chosen by Cargill for the development of their new lactic acid production process (Rush 2012). For reconstruction of complex plant pathways, *S. cerevisiae* is typically preferred over *E. coli*, as it is difficult to express P_450_ enzymes in bacteria, which is illustrated by the production of both resveratrol (Mei et al. 2015) and hydrocortisone (Szczebara et al. 2003) using *S. cerevisiae*. The reconstruction of a plant pathway in yeast was recently taken to the extreme by expressing a 23 enzymatic step pathway for production of opioids (Galanie et al. 2015), but even though the opioids were produced in low levels, it was necessary to engineer the yeast endogenous metabolism to ensure product formation (Galanie et al. 2015). Reconstruction of a biosynthetic pathway in the cell factory of choice is an important step towards industrial production, but as mentioned above, it is subsequently necessary to improve the TRY in order to establish a process. Improving the TRY generally involves many rounds of the DBT cycle and is therefore costly. The reason for this is that metabolism in all organisms has evolved to serve specific purposes, e.g., for yeast to rapidly convert sugars to ethanol in order to capture energy needed for growth and at the same time make a toxic product that prevents growth of competitors. This means that extensive regulatory systems have evolved to ensure that the metabolism is operating robustly, even when the cells are exposed to various perturbations. Due to this tight regulation, it is difficult to redirect the fluxes inside the cell, e.g., to redirect the metabolism from efficiently producing ethanol to producing farnesene.

Whereas the major challenge in developing a new bioprocess is with developing the cell factory, there are also challenges associated with matching the cell factory design with the feedstock that is going to be used for the production. In the bioethanol industry, different yeast strains are used for production in Brazil, where the feedstock is sugar cane (Basso et al. 2008), and in the USA, where the feedstock is primarily corn (Song et al. 2016). Thus, for designing a new bioprocess, it is important to have in mind what the feedstock is going to be for the final bioprocess. The cell factory can be engineered to adapt to alternative feedstock, as illustrated by the extensive engineering of *S. cerevisiae* to use lignocellulosic hydrolysates as a feedstock (Matshushika et al. 2009), and which has now resulted in initiation of second generation ethanol production. Another important aspect to consider is how the fermentation will be integrated with downstream processing and purification of the product. This may have an impact on the cell factory choice and on the design criteria for the cell factory, in particular on how it is going to be tested in each round of the DBT cycle.

## Lower costs, stable production, and lower environmental footprint

For a number of chemicals that have traditionally been produced from petrochemicals or by extraction from plants, the chemical industry has been seeking to establish bio-based production, for primarily the three following reasons: (1) to reduce costs, (2) to ensure stable production, and/or (3) to reduce the environmental footprint. Two of the early examples of this were the development of a process for bio-based production of riboflavin by BASF and of 7-ADCA by DSM. Riboflavin (vitamin B2) is used both as a dietary supplement for humans and in feed, and is therefore produced in large volume. Traditionally, riboflavin was produced through chemical synthesis, but by developing a riboflavin-producing bioprocess through engineering of the filamentous fungus *Ashbya gossypii*, BASF were able to reduce the associated water emissions by 67% and carbon dioxide emissions by 80%, primarily due to reduced energy and water usage (Ledesma-Amaro et al. 2015). 7-ADCA is a precursor for production of the widely used antibiotics cephalexin, and traditionally, 7-ADCA was produced chemically from penicillin. However, by metabolic engineering, a high-yielding strain of *Penicillium chrysogenum*, DSM, established a process for production of adipoyl-7-ADCA that can easily be enzymatically cleaved to adipic acid and 7-ADCA. The bioprocess reduced carbon dioxide emissions by 5% and reduced water and energy usage by 75% and 20%, respectively (Erickson and Hessler 2004).

Many chemicals are obtained from plant sources. Typically, their chemical structure makes them difficult to chemically synthesize and it is therefore cheaper to extract them from plants, even though the yields are very low. A good example of this is artemisinin, which is one of the most efficient antimalarial drugs (WHO 2016). This compound is extracted from *Artemisia annua*, sweet wormwood. There have been large variations in supply, primarily because of the long time between planting and harvest, i.e., farmers have planted extensively when supply was low (and price high), resulting in over-production and dropping prices a few years later. In order to ensure a more stable production, Prof. Jay Keasling and his research group at the University of California Berkeley undertook a large metabolic engineering project on developing a bioprocess for production of artemisinin. Initial work was done in *E. coli* (Tsuruta et al. 2009), but as it was hard to express P_450_ enzymes in this organism, the group switched to yeast as a cell factory (Ro et al. 2006). Following initial development at University of Berkeley, the work was transferred to Amyris, who later partnered with Sanofi-Avensis for industrial production. The research and development work was to a large extent financed by the Bill & Melinda Gates Foundation, who required that the companies involved should sell artemisinin not-for-profit. In 2014, this process reportedly delivering more than 337 million doses of artemisinin-based combination therapies (ACTs), successfully reducing the global malaria burden (WHO 2016). The engineering of yeast for efficient production of the chemical was a major undertaking and involved identification of novel enzymes, engineering of yeast endogenous metabolism and further chemical conversion to obtain the final product (Ro et al. 2006; Westfall et al. 2012; Ro et al. 2008; Paddon et al. 2013). Another plant product for which there has been developed a bioprocess for production is resveratrol. This chemical is an antioxidant present in grapes and it is sold as a dietary supplement. Traditionally, it was extracted from Japanese knotweed, but the company Fluxome reconstructed the resveratrol biosynthetic pathway in yeast, and through further metabolic engineering, a yeast strain was developed that could produce cost-competitive resveratrol resulting in a commercial process (Smits et al., 2011). In 2012, the technology was acquired by Evolva, who is today marketing yeast based resveratrol (Baerends et al. 2014). Resveratrol is derived from either tyrosine or phenylalanine, but it also requires efficient provision of malonyl-CoA and efficient production therefore requires a number of different metabolic engineering steps (Li et al. 2015). Sesquiterpenes are another class of plant metabolites for which there has been much interest in establishing microbial fermentation processes for their production. This is a wide group of chemicals (in the class of isoprenoids) that find many different applications, e.g. perfume ingredients and pharmaceuticals. Through collaboration with Firmenich, we were involved in the early development of a yeast platform for production of sesquiterpenes to be used as perfume ingredients (Asadollahi et al. 2009). The perfume ingredient industry is interested in producing their chemicals using microbial fermentation, as this will enable scalable production and independence from fluctuations in the supply of the plant raw material. Through several rounds of the DBT cycle, we managed to significantly improve the production of α-santalene, normally extracted from sandal wood oil (Schalk and Clark 2007). Today, Firmenich and other companies have partnered with Amyris and/or Evolva for production of many of their perfume ingredients.

Chemical building blocks used for production of plastic polymers are among the chemicals produced and sold in largest volumes. Thus, 1,3 propanediol, traditionally produced by chemical synthesis, used in the production of plastic, detergents, adhesives, composites, antifreeze and heat-transfer industrial fluids coatings, polyurethanes and cosmetics, and 1,4 butanediol, traditionally produced from petroleum-derived feedstock used in the production of plastic, elastic fiber, and polyurethanes are each produced in large volumes. In connection with the development of their new polymer Sorona®, DuPont were, however, interested in developing a bio-based production as this would allow them to reduce costs and also the environmental footprint. Through collaboration with Genencor, they performed extensive metabolic engineering of *E. coli* and thereby developed a cell factory for production of 1,3 propanediol (Nakamura and Whited 2003). In collaboration with Tate & Lyle, they further developed a completely new bioprocess, which was necessary as this was the first large-scale production process using *E. coli* as a cell factory. The development of this process represents one of the milestones in bio-based production as it was the first example of complete metabolic engineering of a cell factory for production of a non-natural-produced chemical combined with development of a novel bioprocess for large-scale production. More recently, Genomatica performed metabolic engineering of *E. coli* for production of 1,4 butanediol (Yim et al. 2011). Through several rounds of the DBT cycle, they obtained an *E. coli* strain that could produce more than 18 g/L in a 140-h fermentation process (Yim et al. 2011). Through partnering with BASF, Novamont, and Cargill, they developed a commercial process for production of 1,4 butanediol, which hereby became the second commodity type chemical produced by microbial fermentation (Burgard et al. 2016).

## New chemicals with desirable properties

The chemical industry is constantly looking for chemicals with new, interesting properties. A classic example of this is the invention of nylon by DuPont in the 1930’s. The very high oil prices around 2008 resulted in the establishment of a large number of projects and biotech companies focusing on production of advanced biofuels, i.e., chemicals that have improved properties compared with ethanol. Ethanol is in many ways an excellent drop-in fuel, but it does have some drawbacks: (1) its energy content is quite low and (2) it is hydroscopic, i.e., it attracts water and it is therefore not suitable for use in boat engines, and this also makes it unsuitable for transport via pipelines. There has therefore been much interest in producing butanol, which has a higher energy density and is less hydroscopic than ethanol, and is therefore an ideal drop-in fuel in gasoline. Furthermore, isobutanol can easily be chemically converted to isobutylene, which is used to make isooctane, isooctene, and jet fuel. This resulted in two large initiatives for production of isobutanol, one driven by Butamax, a joint venture between DuPont and BP, and the other driven by Gevo (Hong and Nielsen 2012). Both companies performed extensive metabolic engineering of yeast in order to develop a cell factory that could meet the TRY requirement for production of isobutanol, and currently Gevo is producing isobutanol commercially. Another large initiative has been driven by Amyris, who used their yeast platform strain for production of farnesene, which is a sesquiterpene derived by a single enzymatic step from the endogenous yeast metabolite farnesylpyrophosphate (Hong and Nielsen 2012; Tippmann et al. 2016). In 2010, Amyris partnered with Total on development of this process, and even though the yeast cell factory is still being further improved, Amyris is producing farnesene to be used as jet fuel and as a drop in fuel in diesel for busses in Brazil.

In the last couple of years, there has been much focus on production of other types of hydrocarbons, in particular alkane and alkenes. This was initiated by the identification of the enzymes responsible for the conversion of fatty acids to alkanes and reconstruction of these in *E. coli* (Choi and Lee 2013). Today many more enzymes have been discovered (Kang and Nielsen 2016) also allowing for production of short chain alkanes/alkenes in *E. coli* (Choi and Lee 2013). Recently alkane production was also demonstrated in *S. cerevisiae* (Buijs et al. 2015; Foo et al., 2017) and through engineering of the fatty acid production, it was demonstrated possible to improve the production of alkanes/alkenes significantly (Zhou et al. 2016).

As mentioned above, another example of a new chemical introduced is polylactic acid (PLA), a polymer derived from lactic acid. Polymer chemists at Cargill identified a route for production of PLA, in which a very pure form of the L-isomer is required for production of polylactic acids with high melting point and high crystallinity (Lunt 1998). Lactic acid, which is used in the food industry for preservation, is traditionally produced by fermentation of lactic acid bacteria (LAB), but these cell factories require complex media as they are auxotrophic for several amino acids. It is therefore costly to obtain a pure form of lactic acid and this led Cargill researchers to look for an alternative cell factory that could grow on a minimal medium. They added the ability to grow and produce lactic acid at low pH as an additional criterion, as this would allow them to produce lactic acid directly and not lactate, as produced by LAB. As it is the lactic acid that is required for polymerization, the use of lactate would require a conversion to lactic acid through addition of an inorganic acid and this would result in production of 1.2 kg of gypsum per kilogram of lactic acid produced. Their search resulted in the identification of a low pH-tolerant yeast species that they could eventually engineer to efficiently produce lactic acid and convert this further to PLA (Rush 2012). This resulted in establishment of a 140,000-metric tons/year production plant of PLA in Blair, Nebraska, USA (Nature Works LLC 2005). This case is interesting as it is a clear demonstration that it may be beneficial to use a non-conventional cell factory for a new bioprocess. It required significant investments by Cargill to develop the genetic tools for metabolic engineering of their yeast strain, but they are now offering the yeast strain together with tools as a technology package for other companies interested in producing organic acid. This has recently been exploited by BioAmber for production of succinic acid (Hartmann 2010), which is of much interest as a chemical building block for a variety of polymers, that is, bio-based succinic acid can be transformed into 1,4-butanediol (BDO) and tetrahydrofuran (THF) in a single catalytic step (Cavani et al. 2016).

## Harnessing the biodiversity of the Red Sea: Transition to renewable resources

The Middle East countries currently have a largely oil-dependent industry and infrastructure, which results in very high carbon emissions per capita; e.g. 55 tons per capita in the United Arab Emirates, compared to only 22 tons per capita in the US (Zafar 2017). To change this trend, the region is anticipating investments in renewable energy in the order of $35 billion per year by the year 2020 (Zafar 2017). However, many countries in the region have severe limitations in producing biomass to replace oil, simply because the arable land is very scarce. For example, Saudi Arabian territory is mostly desert, and only 1.5% of the land is arable (Crop Trust 2017). A viable alternative for such countries is to turn to photosynthetic sea organisms for biomass production and engineering of cell factories.

The Red Sea is a prime example of such an alternative bio-resource. It stretches approximately 2250 km in length, but it is largely an isolated body of water, as the southern opening is merely a narrow passage to the Indian Ocean and there are no rivers flowing into it. This desert bound sea exhibits consistently high salinity gradient (36–41 p.s.u) owing to high surface evaporation in the north, and maintains a uniform temperature of 21.5 °C below the heated (28–32 °C) surface layer. Consequently, this body of water is uniquely warmer, has low nutrient content and is hypersaline with more than 10% of the fish species being endemic (Di Battista et al. 2013). It is an implied evolutionary incubator that occasionally exports species into the adjacent Indian Ocean (Di Battista et al. 2013). The diverse Red Sea ecosystem includes reef sharks, dolphins, turtles, stingrays, sea urchins, sea cucumbers and a huge variety of corals, sponges, mollusks and smaller fish species. All these depend on the hypersaline adapted bacteria and archaea to maintain ecological balance of the reef. This particular ecological niche represents an untapped potential for developing efficient cell factories along two main axes (Fig. 1). Firstly, it could be expected to provide access to new salt-tolerant, thermotolerant and organic solvent-tolerant enzymes and specific metabolic pathways that evolved in this particular environment (Khandeparker et al. 2011; Xin and Hui-Ying 2013; Yu and Li 2014). Those features are particularly interesting for industrial processes, and could significantly expand our synthetic biology toolbox. Secondly, the ability of local organisms to grow in a high salinity environment could lead to isolation of saline-tolerant cell factories that can be used for biosustainable production of fuels and chemicals using saline water as a source for production. This would considerably reduce the risk of contamination and bypass the requirement for desalination, which is extremely expensive and generates a large environmental footprint. Under the economic diversification plan “Vision 2030”, Saudi Arabia aims to take a regional lead in the process of developing new technologies for bio-based production of high value chemicals by Red Sea microorganisms. The King Abdullah University of Science and Technology (KAUST) therefore runs a broad research program aiming to deliver the key enabling technologies for this transition to bio-based economy. In the previous sections we have outlined and discussed the key global drivers for the shift to bio-based production: expanding the range of molecules produced by cell factories, and reducing the price and the environmental footprint of the production. The main challenges we outlined are the correct choice of the cell factory, development of engineering tools, and matching the desired product with access to feedstock and the metabolic capacity of the cell factory. In the following sections, we will discuss how these particular challenges could be addressed in the region, using the biodiversity potential of the Red Sea. We will focus on screening for the most promising cell factories that can be derived from the local environment, and production processes that match the metabolic setup of the local cell factories and the available carbon sources.

## Hypersaline adapted bacteria inhabiting the Red Sea

The unique Red Sea ecosystems epitomize huge opportunities for successful identification of novel biocatalysts, as well as microbial cell factories. Consequently, diverse Red Sea associated microbiomes have been the focus of several recent studies at KAUST, including those from different environments: mangrove sediment (Al-Amoudi et al. 2016a; Al-Amoudi et al. 2016b; Alzubaidy et al. 2016; Simões et al. 2015), sea water (Qian et al. 2011; Jimenez-Infante et al. 2016), coral (Bayer et al. 2013), sponge (Gao et al. 2015; Tian et al. 2014), and extreme niches such as Red Sea brine pools (Mwirichia et al. 2016; Zhang et al. 2015; Kamanda Ngugi et al. 2015; Siam et al. 2012; Abdallah et al. 2014; Antunes et al. 2015; Behzad et al. 2016; Jimenez-Infante et al. 2014; Grötzinger et al. 2014). Such studies focused on relevant microbial bioactivities, such as for example antibacterial activity (Sagar et al., 2013a, b; Moitinho-Silva et al. 2014; Abdelmohsen et al. 2014). Metagenomics studies of Red Sea samples show a distinctive composition of local microbiota, quite distinct from other marine environments (Thompson et al. 2013). This is a specific consequence of the 25 Red Sea brine pools, thought to be inhospitable to organic life, harboring bacteria that thrive in these hydrothermal and hypersaline deep-sea environments (Mwirichia et al. 2016; Zhang et al. 2015; Kamanda Ngugi et al. 2015; Grötzinger et al. 2014; Siam et al. 2012; Abdallah et al. 2014; Antunes et al. 2015; Behzad et al. 2016; Jimenez-Infante et al. 2014). An interesting study of vertical stratification was reported for two hydrothermal and hypersaline deep-sea pools in the Red Sea: the Atlantis II and Discovery. A metagenomic study of the two deep-sea pools showed that alpha-Proteobacteria dominated the deep sea, Planctomycetaceae, or Deferribacteres dominated the brine-seawater interfaces, while gamma-Proteobacteria dominated brine pools (Bougouffa et al. 2013). Multivariate analysis of the data indicated that temperature and salinity were the two major influences shaping the composition of these communities. From the metagenome analysis, a novel mercuric reductase was derived, which was collected from the lower convective layer (LCL) of the Atlantis II (ATII) deep brine pool (Sayed et al. 2014). The amino acid sequences for this ATII-LCL mercuric reductase differs from the soil derived orthologs by only <10% but exhibits striking functional differences as it is in vivo functional in high salt, stable at high temperature, efficiently detoxifies, and is resistant to increased levels of Hg^2+^ in vivo (Sayed et al. 2014). This indicated that the Red Sea extremophiles have developed specific metabolic pathways and enzymes to cope with their environment, and they can clearly serve as a source for potential biotechnology-based applications. Genomes of several microorganisms with unusual features were also isolated and sequenced, such as *Candidatus* Synechococcus spongiarum (Gao et al. 2014), a sponge symbiont, and several extremophiles isolated from the deep-sea anoxic brine lakes, including *Haloplasma contractile* (Antunes et al. 2011a), *Halorhabdus tiamatea* (Antunes et al. 2011b), and *Salinisphaera shabanensis* (Antunes et al. 2011c). *Haloplasma contractile* was reported to represent a new order situated between Firmicutes and Mollicutes (Antunes et al. 2011a). This bacterium has an unusual morphology with contractile protrusions. In line with this observation, its genome harbors an unusually high number of MreB/Mbl homologs that are known to play a role in cell morphogenesis. *Halorhabdus tiamatea* is the first archaeon isolated from deep-sea anoxic brine lakes (Antunes et al. 2011b). A genome comparison with *Halorhabdus utahensis* suggests that *H. tiamatea* has significantly more transmembrane transport genes and a putative trehalose synthase and lactate dehydrogenase (LDH). Trehalose synthase has never been detected in other members of Halobacteriaceae. Also, no LDH homologs had been detected in haloarchaeal genomes, even though LDH activity has been reported in *Halobacterium salinarum* cell extracts (Bhaumik and Sonawat, 1994).

Since most of the downstream experiments conducted on isolated strains critically depend on findings in the initial metagenomic screens, and because the detailed functional annotation of newly sequences microbial genomes is time-consuming due to integration of variety of experimental and computational data, a pipeline for Automatic Annotation of Microbial Genomes (AAMG) was developed (Alam et al. 2013). This annotation pipeline can be accessed through INDIGO web server (freely available at http://www.cbrc.kaust.edu.sa/indigo). INDIGO is a data warehouse system that not only provides integration of annotations for exploration of information but also enables detailed analysis of newly sequenced microbial genomes.

## Identification of novel cell factories from the Red Sea isolates

As discussed above, the choice of the cell factory and matching the desired product to the metabolic makeup of the cell factory are the key challenges for developing bio-based production. The efforts in this direction started with large screening initiatives, focused on finding potential cell factories in environmental samples from the Red Sea. The first study that compared Red Sea lagoon microbiomes in terms of diversity, taxonomy, and antimicrobial effects was finalized in 2016 (Al-Amoudi et al. 2016b). The study explored mangrove mud and the microbial mat sediments collected from the Rabigh harbor lagoon (RHL) and Al Kharrar lagoon (AKL) for antimicrobial bioprospecting. Results suggested that the mangrove mud samples are superior in terms of taxonomical abundance, species diversity, percentage of DNA sequences for enzymes associated with antibiotic synthesis, polyketide synthases (PKS), and nonribosomal peptide synthetases (NRPS), compared to microbial mat samples from salt marsh. Moreover, the antibiotic biosynthesis activity related to PKS or NRPS was higher in Firmicutes (Bacilli and Clostridia) compared to other phyla. Interesting conclusions could be drawn when correlating the community composition of the mangrove mud samples with hydrocarbon content. The samples from the non-industrialized AKL site show a shift in dominant phyla consistent with early hydrocarbon contamination exposure, similar to those reported for the Prestige oil spill metagenomic samples collected in 2004, which was dominated by Proteobacteria (primarily comprised of gamma- and delta-Proteobacteria) (Acosta-Gonzalez et al. 2013). The mangrove mud samples from the industrialized RHL site exhibit a shift in terms of dominant phyla consistent with late hydrocarbon contamination exposure, similar to the Prestige oil spill metagenomic samples collected in 2007 (Acosta-Gonzalez et al. 2013). The correlation to exposure to hydrocarbon contamination highlighted an interesting feature of the proportion of Firmicutes, which is highest in the pristine mangrove mud (Alzubaidy et al. 2016), followed by the AKL mangrove mud, and lowest in the RHL mangrove mud. This indicated that the more robust Firmicutes (Bacilli and Clostridia) with potential to produce secondary metabolites of interest can be cultured from the RHL mangrove mud samples.

To experimentally screen for the in silico detected potential for synthesis of antimicrobials, traditional culturing methods were applied (Al-Amoudi et al. 2016a). Screening the cultured strains for antimicrobial activity confirmed the in silico detected presence of PKS and NRPS, since most of the positive screening results were from Firmicutes (Al-Amoudi et al. 2016a). Moreover, 10 of the strains displayed zone inhibition against all three-indicator used pathogens, of which nine strains belong to the phylum Firmicutes, while one belongs to the phylum Proteobacteria. Since PKS and NRPS have been found to support synthesis of secondary metabolites that act as antibiotics, immunosuppressants, toxins, siderophores, or antitumor agents (Amoutzias et al. 2016), their presence increases chances of identifying organisms capable of producing the abovementioned bioactive secondary metabolites. For the 10 strains displaying zone inhibition against all three-indicator pathogens, PKS and/or NRPS gene sequences were found to be present in strains belonging to species *Bacillus licheniformis*, *Bacillus vallismortis*, *B. subtilis*, and *Paenibacillus dendritiformis*. The remaining strains (belonging to species *Bacillus sonorensis*, *Brevibacillus borstelensis*, and *Aneurinibacillus migulanus*) did not have identifiable PKS and/or NRPS genes and thus possibly harbor novel biosynthetic clusters with capacity to synthesize antibacterial compounds. Some of these species may very well serve as completely novel cell factory platforms in the future, where sea water can be used for low-cost bioproduction of bioactive compounds.

From the abovementioned samples, several amylase and lipase enzymes were identified in both the AKL and RHL metagenomic datasets; most of which were derived from Bacilli (Al-Amoudi et al. 2016b). Amylases and lipases are important industrial biocatalysts used in several industries that require thermostable enzymes suitable for starch degradation and production of glucose, maltose, and dextrose (Asgher et al. 2007; Gomes et al. 2003), as well as enzymes that remain active in industrial processes where concentrated salt solutions are used (Prakash et al. 2009). Halobacterial enzymes are found to be thermotolerant and stable at room temperature for extended periods (Mohapatra et al. 1998). Several thermostable α-amylase have been derived from Bacilli strains such as *Bacillus stearothermophilus* and *B. licheniformis* (used in starch processing industries) (Gomes et al. 2003), as well as halophilic amylases derived from *Chromohalobacter* sp. (Prakash et al. 2009), *Halobacillus* sp. (Amoozegar et al. 2003), *Halomonas meridiana* (Coronado et al. 2000), and *Bacillus dipsosauri* (Deutch 2002). *Bacillus* is also one of several microbial genera used for the commercial production of lipases (Aravindan et al. 2007). The search for thermostable lipases continues, and several such thermostable lipases have been isolated and characterized primarily from Bacilli (Kumar et al. 2014; Kumar et al. 2005; Castro-Ochoa et al. 2005). Owing to the extreme characteristics of these Red Sea lagoons, Bacilli strains from these environments could be expected to be extremely robust in other extreme environments, including high density production conditions, since they demonstrated tolerance in ecosystems exposed to hydrocarbon contamination. They could be expected to harbor the halophilic thermostable amylases and lipases required by industry as biocatalysts. The overall outcome of these screening initiatives suggests that the local Bacilli represent an important source of industrially relevant enzymes and new antimicrobials, and their development into cell factories could be a plausible route for bio-based production in the region.

## Cyanobacteria: the potential to derive biomass from solar energy

The remaining important challenge in developing bio-based processes is the feedstock availability. As discussed above, with very limited arable land in the region calls imposes drastic restrictions on producing terrestrial plant biomass. Photosynthetic sea organisms should thus be a preferred choice. Cyanobacteria are very abundant photosynthetic bacteria, with a widely recognized potential to produce secondary metabolites and biofuels (Cassier-Chauvat et al. 2017). They have a complex metabolism, and the regulatory mechanisms that coordinate the light harvesting module with the biosynthetic modules have been extensively modeled in silico (Westermark and Steuer 2016). Diversity of hydrocarbons produced by Cyanobacteria in terms of chain length, degree of saturation, and variations of the carbon skeleton has been highlighted by Xie et al. (2017). Considerable efforts have been directed to metabolic engineering of Cyanobacteria, mainly geared towards biofuel production (Al-Haj et al. 2016; Oliver et al. 2016; Johnson et al. 2016; Gomaa et al. 2016). The major optimization strategies were directed towards improving carbon fixation (Atsumi et al. 2009), improving the capture of the light energy (Iwaki et al. 2006), and improving product extraction by the use of induced autolysis systems (Miyake et al. 2014).

With the global horizontal irradiance ranging between 5700 to 6700 Wh/m^2^, Saudi Arabia has one of the highest solar energy potentials on the planet. On the other hand, the scarcity of fertile soil limits the availability of plant biomass, which is the basic resource for this type of biotechnology worldwide. Cyanobacteria, with their capacity to capture solar energy and convert CO_2_ to sugars, and ultimately biomass, present a promising solution to bridge this gap. The major hurdle that has to be overcome is the relatively slow growth of Cyanobacteria, compounded by the fact that simple ad hoc strategies to improve product yield have been largely unsuccessful (Ruffing 2013; Liu et al. 2011; Oliver et al. 2016; Wang et al. 2013). To overcome this hurdle, more comprehensive metabolic optimization strategies are needed to guide the engineering of Cyanobacteria for increased growth rate and biomass production. The KAUST research program for developing new technologies for bio-based production has isolated a large number of Cyanobacterial species from the local environmental samples that show potential as producers of biomass. They can be potentially used for production or animal and fish feed, and those that have advantageous properties of lipid metabolism could be geared towards the production of biofuels. To facilitate the use of these isolates, KAUST has developed a computational framework to guide genome reduction of cyanobacterial strains with the aim of generating the so-called “clean” genomes that retain all genetic components necessary for the organism to survive and yet remain able to sustain production of desired chemicals. Additionally, the reduced genome is expected to have improved genome stability and possibly an improved growth rate. It should be noted that some Cyanobacteria possess small genomes but exhibit high ploidy level that is growth phase-regulated (Greise et al. 2011), and this should be taken into account when genome reduction is attempted. The key feature of this computational framework is that it guides genome reduction and does not require the availability of a sequenced genome of a closely related strain. Thus far, genomic islands and prophage components that could be eliminated from genomes of the strains from cyanobacterial genus *Synechococcus* have been identified (Bajic VB and Essack M, unpublished results). The same approach was applied to the Bacilli isolated from the Red Sea: *Bacillus foraminis*, *B. licheniformis*, *Bacillus amyloliquefaciens*, *Bacillus niabensis*, *B. vallismortis*, and *Bacillus acidicola* strain. The strategy chosen was inspired by Posfai et al. (2006) who demonstrated that the reduction of the *E. coli* K-12 MG1655 genome by ∼15% via genetic engineering (aimed to obtain the “clean” genome) achieved a 83% L-threonine overproduction compared to the wild-type strain.

Proof-of-concept studies exist for using Cyanobacteria as producers of biofuels (Lindberg et al. 2010; Oliver et al. 2013; Nozzi et al. 2013) or bioactive compounds (Singh et al. 2005; Essack et al. 2014; Singh et al. 2016; Humisto et al. 2016). However, the currently available engineered strains are not producing sufficient amounts of free fatty acids (FFA) to be commercially viable. Attempts have been made to commercialize ethanol production from Cyanobacteria, and some of these technologies have been patented by Algenol Biofuels and Joule Unlimited, reviewed by Dexter et al. (2015). The main challenge in this process has been the dilute rate of ethanol production, hampering the economic competitiveness of the process. Despite these difficulties, the US Environmental Protection Agency has approved a renewable fuel pathway developed by Joule Unlimited, allowing the company to commercialize biofuels using its Cyanobactyeria-derived ethanol (Voegele 2016).

In order to guide further improvement strategies, we exploited the availability of over 120 cyanobacterial genomes sequenced to-date, to establish a computational pipeline for evaluating their natural potential to produce and secrete FFA. Thus, we have developed an FFA SCreen (FFASC) tool, to evaluate potential for FFA production and secretion by cyanobacterial strains based on their theoretical proteomes (Motwalli et al. 2017). Our results strongly suggest that the top-ranked cyanobacterial strains that should be targeted for this purpose primarily include *Prochlorococcus* (order Prochlorales) and marine *Synechococcus* (order Chroococcales). Another interesting application of Cyanobacteria as cell factories is in the production of metabolites that are toxic to both terrestrial and aquatic invertebrates, and could serve as potential pesticides (Essack et al. 2014). Cyanobacterial genera we identified in this process as potential producers of pesticides include *Lyngbya*, *Leptolyngbya*, *Phormidium*, *Nostoc*, *Microcystis*, and *Planktothrix*. It is interesting to note that among the Cyanobacteria with top ranking as FFA producers, there are some nonphotosynthetic organisms. The cyanobacterial endosymbiont of *Epithemia turgida*, isolate EtSB Lake Yunoko (Nayakama et al. 2014) ranked at position 1, and *Candidatus* Atelocyanobacterium thalassa (isolate ALOHA) (Tripp et al. 2010) ranked at position 7. Both these strains do not have the necessary machinery to conduct photosynthesis due to some form of evolutionary metabolic streamlining. This streamlining could possibly have enhanced their ability to produce FFA, in a manner similar to reported genome reduction outcomes (Pósfai et al. 2006; Baumgart et al. 2013; Morimoto et al. 2008). Both these strains are symbionts of photosynthetic diatoms and algal strains that are generally known to produce FFA (Sharma et al. 2012). Potentially, these two organisms open different venues for engineering Cyanobacteria for FFA production, through a symbiotic combination of microorganisms. To resume, local Cyanobacteria have the potential to secure feedstock for bio-based production, and their metabolic makeup is especially suited for production of FFA, and possibly other types of biofuels. The response of different species of Cyanobacteria to high irradiation varies (Dillon et al. 2003), but there are production strains, such as *Synechococcus* sp., which are highly tolerant to high irradiation at atmospheric levels of CO_2_ (Bernstein et al. 2016). One of the major challenges in developing Cyanobacteria-based, non-sterilized outdoor cultivations will be possible contaminations with other microorganisms living in the Red Sea (Raitsos et al. 2013). Strategies have been developed to eliminate specific contaminants of cyanobacterial outdoor cultivations, such as the bicarbonate-based pH-rising strategy (Zhu et al. 2017). Along these lines, specific solutions will have to be developed to handle the local contaminants.

While they represent a very promising venue, Cyanobacteria are not the only possible route. High levels of sun radiation in the region could also be used to power solar panels to generate reducing power on large cathodic surfaces (Lovley and Nevin 2013). Solar energy could thus be used to power the production of various carbon compounds in non-photosynthetic bacteria (Liu et al. 2015; Sakimoto et al. 2016), thereby expanding the range of available cell factories.

## Conclusions and perspectives

We have argued that the rapid expansion of bio-based production of fuels and chemicals by microbial cell factories is driven by the three major factors: (i) the versatility of microbes that can produce new molecules with improved properties compared with currently available ones, (ii) the ability to produce fuels and chemicals cheaper than the traditional chemical synthesis, and (iii) the opportunity to produce chemicals with a reduced environmental footprint. The Red Sea organisms, adapted for survival in a unique environmental niche, can be successfully exploited along all three of these axes. We have discussed the available results from the ongoing studies that clearly indicate that the bioprospecting in the Red Sea can deliver significant advances in terms of versatility of produced compounds. The tolerance of high salinity and the ability to perform photosynthesis in this highly irradiated area also provide a solid basis for reducing the cost of fermentation and the environmental footprint, be bypassing the need for an additional source of carbon, desalination or sterilization. The future progress should obviously focus on extensive marine bioprospecting based on metagenomics approaches (Kodzius and Gojobori 2015). Sample heterogeneity is a major issue when dealing with environmental omics, and single cell techniques for reducing sample heterogeneity are arguably the best way forward (Kodzius and Gojobori 2016). These experimental approaches should be followed by comprehensive in silico comparison of metabolic features of local species compared to their counterparts from other environments. This should be used to pin-point distinguishing features that could ear-mark new species for development of cell factories, or simply as “donors” of new pathways for metabolic engineering of existing cell factories. Efficient in silico tools for prioritizing among many available candidates species in the natural isolates are of paramount importance in driving this process. Finally, developing genetic tools for engineering the local species will be the final requirement on the way to establishing a competitive bio-based production in the region.
